# Advantages of ketamine in pediatric anesthesia

**DOI:** 10.1515/med-2022-0509

**Published:** 2022-07-06

**Authors:** Alessandro Simonini, Etrusca Brogi, Marco Cascella, Alessandro Vittori

**Affiliations:** Department of Pediatric Anaesthesia and Intensive Care, S.C. SOD Anestesia e Rianimazione Pediatrica, Ospedale G. Salesi, Ancona, 60123, Italy; Department Anesthesia and Intensive Care, University of Pisa, Pisa, 56126, Italy; Department of Supportive Care, Division of Anesthesia and Pain Medicine, Istituto Nazionale Tumori, IRCCS Fondazione G. Pascale, Naples, 80100, Italy; Department of Anesthesia and Critical Care, ARCO Roma Ospedale Pediatrico Bambino Gesù IRCCS, Piazza S. Onofrio 4, 00165, Rome, Italy

**Keywords:** ketamine, pediatrics, sedation, anesthesia, intensive care

## Abstract

Although ketamine is primarily used for induction and maintenance of general anesthesia, it also presents sedative, amnestic, anesthetics, analgesic, antihyperalgesia, neuroprotective, anti-inflammatory, immunomodulant, and antidepressant effects. Its unique pharmacodynamics and pharmacokinetic properties allow the use of ketamine in various clinical settings including sedation, ambulatory anesthesia, and intensive care practices. It has also adopted to manage acute and chronic pain management. Clinically, ketamine produces dissociative sedation, analgesia, and amnesia while maintaining laryngeal reflexes, with respiratory and cardiovascular stability. Notably, it does not cause respiratory depression, maintaining both the hypercapnic reflex and the residual functional capacity with a moderate bronchodilation effect. In the pediatric population, ketamine can be administered through practically all routes, making it an advantageous drug for the sedation required setting such as placement of difficult vascular access and in uncooperative and oppositional children. Consequently, ketamine is indicated in prehospital induction of anesthesia, induction of anesthesia in potentially hemodynamic unstable patients, and in patients at risk of bronchospasm. Even more, ketamine does not increase intracranial pressure, and it can be safely used also in patients with traumatic brain injuries. This article is aimed to provide a brief and practical summary of the role of ketamine in the pediatric field.

## 1 Introduction

Given its respiratory and cardiovascular stability, ketamine is considered an ideal anesthetic drug, especially in prehospital and emergency department settings [1]. Furthermore, due to the peculiarity of its pharmacological properties, this drug presents several potential applications, such as sedation during pediatrics procedures, acute and chronic pain management, ambulatory anesthesia, and intensive care practices [2,3]. Ketamine has hypnotic, amnesic, analgesic, anti-inflammatory, and sympathomimetic properties and determines dissociative anesthesia while maintaining laryngeal reflexes, with respiratory and cardiovascular stabilities [4]. Remarkably, ketamine does not cause respiratory depression, maintaining both the hypercapnic reflex and the residual functional capacity with a moderate bronchodilation effect [5].

Besides the many advantageous properties, ketamine is burdened by undesirable side effects related to its psychomimetic (i.e., delirium, psychosis, perceptual, and thought disorders with hallucinations) and sympathomimetic actions (i.e., arterial, and intraocular hypertension, airway mucus hypersecretions) [6,7]. Conversely, psychomimetic reactions during recovery are rare, transient, and generally not disturbing in children. The incidence of severe recovery agitation is about 1.4% [8,9]. The administration of benzodiazepines to overcome the psychomimetic effects of ketamine is widely used by practitioners. Nevertheless, it is still controversial, and the optimal midazolam doses are not already confirmed [10,11,12,13]. Generally, ketamine is not used in psychiatric patients due to the theoretical risks of exacerbating psychosis in such patients; however, evidence is arising that a single dose of ketamine should not exacerbate psychiatric symptoms [14,15].

Even more, ketamine has shown some interesting anti-inflammatory, immunomodulant, and antidepressant properties that warrant further research [6].

A very important concern is the theoretical risk of apoptosis and neurodegeneration caused by drugs used to provide sedation, analgesia, and anesthesia for infants and young children (particularly those under the age of 3 years). In this regard, in the last years, a flourishing debate is grown on the possible neuroprotective and proapoptotic effects of ketamine [16], mostly about its dose-dependent neurodegenerative effect [17]. Most the anesthetic drugs have been associated with an increase in the rate of apoptosis in laboratory animals [17,18]. Consequently, some fears are raised concerning the use of these drugs in pediatrics. However, at the same time, inadequate pain-control measures are associated with long-term sequelae [19]. Up to now, preliminary data focused on anesthetics drugs are inconclusive, and it is not possible to draw any conclusion regarding a safety margin for the use of ketamine *in vivo* compared to other anesthetics currently used in the clinical setting [17]; this aspect is worth to evaluate deeply in further *in vivo* and *in vitro* trials [20,21].

Despite these undesirable properties, ketamine still represents a drug with a lot of potential in the practice of pediatric anesthesia. In this article, we aimed to provide a brief and practical summary of the role of ketamine in the pediatric field.

### 1.1 Pharmacokinetics and pharmacodynamic notes

Ketamine is a noncompetitive agonist of the *N*-methyl-d-aspartate (NMDA) receptor and inhibits the glutamate activation of the channel; consequently, ketamine inhibits the excitatory effects of glutamate on the CNS [22]. Even more, ketamine inhibits the voltage-dependent sodium channels (hypnosis and local anesthesia effect), blocks the acetylcholine receptors (bronchodilator effect), and interacts with opioids receptor, adenosine receptor, and purinergic receptor. Due to its lipophilicity, the drug crosses easily the blood–brain barrier.

Ketamine can be administered through practically all routes, although the intravenous and intramuscular ones are by far the most used especially for sedation purposes, in uncooperative patients and in emergency settings [23,24]. Since this drug is poorly bound to plasma proteins (10–30%), in the child, who has a peculiar and variable body composition based on the age group, ketamine presents a lower volume of distribution, in comparison to the adult; it is rapidly active (distribution half-life 7–11 min) with short elimination half-life (2–4 h) [6]. When intravenously administered, the onset of action ranges from 30 to 40 s, and the average duration of action is short (5–10 min) with a mean recovery period of 60–90 min. In the case of longer procedures, further incremental doses of 0.5–1.0 mg/kg can be administered intravenously [1]. Intramuscularly, ketamine is absorbed from 90 to 93%, and the time to onset of effects can be reached in 5 min with a mean recovery period of 90–150 min; the duration of effective dissociation after a single dose via this route is of about 20–30 min [6]. In addition to the intravenous and intramuscular routes, ketamine can be administered rectally, orally, or intranasally [25].

Ketamine is metabolized by the liver within the P_450_ cytochrome system and accumulated in the body fat during a continuous infusion. It is mainly excreted (almost 90%) in the urine and by 5% is recovered in feces [1].

The pediatric peculiarities make the kinetics of drugs less predictable than in the adult patient due to the incomplete maturation of organs and systems in the first periods of life. In particular, at birth and in the first months of life, liver function is reduced and characterized by an immature glucuronic-conjugation system and inefficient albumin synthesis [26,27]. Even more, body water composition varies widely during growth, influencing the distribution of the drugs [28].

An overview of proposed dosages of ketamine and bioavailability is presented in Table 1. A summary of the potential applications of ketamine in children care is presented in Table 2.

**Table 1:** Proposed dosages of ketamine and bioavailability

Route of administration	Starting dose (mg/kg)	Bioavailability (%)
Intravenous	0.25–2°	100
	1–2*	
Intramuscular	4–5°	93
	8–10*	
Intraosseous	0.5–2°	100
	1–2*	
Rectal	8–10*	25–30
Oral	6–10°	16–20
Nasal	0.25–4°	45–50
	3–9*	

Legend: °Analgesia and sedation; *Anesthesia dose.

**Table 2:** Summary of the potential applications of ketamine in children care

Clinical use	Notes	Doses	Ref.
Premedication	Alone or in combination with midazolam. Mostly in children with difficult venous access or when venous access is required for induction to provide better conditions for the insertion of a venous catheter	4–6 mg/kg (orally)	[37–41]
		2–4 mg/kg (im)	
Placement of the venous access	Ketamine plays a key role in the sedation of children undergoing various procedures in different clinical settings	5 mg/kg (orally)	[25,36,42]
Caudal regional anesthesia	In addition to local anesthetic, ketamine may prolong postoperative pain relief with minimum adverse effects compared with local anesthetic alone. Animal model showed that in doses commonly used for regional anesthesia, it did not show neurotoxic effect	0.5 mg/kg	[94–98]
Pediatric non-operating room anesthesia	Ketamine has rapid action, short duration, safety profile, possible administration by almost any route, and sedative/analgesic effects. It can be used in combination with other sedatives (e.g., propofol)	0.5 mg/kg (ev)	[79–84]
		Other routes allowed	
Upper respiratory tract infections	Prevention of airway reactivity during the perioperative period	4–6 mg/kg (orally)	[61–69]
		2–4 mg/kg (im)	
General anesthesia in patients with special needs	Induction of anesthesia in potentially hemodynamic unstable patients, induction of anesthesia in patient at risk of bronchospasm, neuromuscular disorders, and at risk of malignant hyperthermia	1–2 mg/kg (ev)	[74–76]
Difficult airways	Effective sedation for fiber-assisted intubation. The sympathomimetic properties can be counterbalanced by the sympatholytic effects of dexmedetomidine	4–6 mg/kg (orally)	55–60
		2–4 mg/kg (im)	
Rapid sequence intubation	It allows rapid onset of action, short duration of action, effective and reliable in producing adequate sedation, maintaining laryngeal reflexes, with respiratory and cardiovascular stability	1–2 mg/kg (ev)	[1,45–54]
	Specific concerns include the possible increase in intracranial pressure, intraocular pressure, myocardial depression, and psychotomimetic effects		
Analgesia	It is a NMDA receptor antagonist, inhibits nitric oxide synthase, and acts via opioid receptors (opioid-sparing effect, and prevention of hyperalgesia phenomenon). It can be used for the prevention and treatment of perioperative pain and pain not related to surgery	5 mg/kg, 0.1 mg/kg/h (intranasally)	[22,99,102–113]
Prevention of emergence delirium	Several studies reported that the use of ketamine can significantly prevent the occurrence of the complication	0.25 mg/kg (iv)	[121–126]
		5 mg/kg (orally)	
Pediatric intensive care	Sedation of mechanically ventilated patients. It maintains lung compliance by reducing airway resistance (e.g., in children with asthma). Beneficial effects on the cardiovascular system	0.2–6 mg/kg/h	[131–136]

### 1.2 Racemic ketamine vs *S*-(+)-ketamine

Chemically, ketamine is an enantiomeric, lipid-soluble phencyclidine derivative. It consists of two optical isomers, namely, *S*- and *R*-ketamine [24]. Ketamine is now available in racemic and pure form. The chiral form consists of two optical isomers, namely, *S*- and *R*-ketamine [24]. The pure dextrorotatory enantiomer of ketamine, *S*(+) ketamine, shows fourfold greater affinity for the NMDA receptor than the *R*-ketamine [29]. Even more, *S*(+) ketamine presents a four-time higher anesthetic and analgesic potency than *R*(–) ketamine. Consequently, lower dosages are needed with minor psychotomimetic side effects, compared with *R*-ketamine [30].

The pharmacological properties of the *S*-enantiomer are comparable to those of the racemic mixture although the analgesic and hypnotic power of the *S*-enantiomer is approximately double that of the racemic. The *S*-(+)-ketamine determines a faster induction of anesthesia with fewer adverse reactions compared to the racemic. *S*-(+)-ketamine showed a faster recovery of psychomotor functions after anesthesia than for *R*-(−)-ketamine [31]. The higher anesthetic potency and lower psychotomimetic adverse effects suggest that *S*-(+)-ketamine has a higher therapeutic efficacy than the racemic mixture [32]; consequently, the pure enantiomer as a pharmaceutical preparation has been widely used nowadays.

After the administration of racemic and *S*-(+)-ketamine, the evaluation of cardiovascular parameters in young healthy volunteers was similar; however, *S*-(+)-ketamine offered clear advantages as an anesthetic drug, with a significant improvement in recovery times and a quantitative reduction of the dose administered [33]. There is also evidence that *S*-(+)-ketamine offers up to 50% better performance in terms of faster recovery of cognitive performance, greater acceptance by healthy volunteers, and identical depth of anesthesia after injection, compared to racemic ketamine [34]. There is evidence that subanesthetic doses of *S*-(+)-ketamine produce hallucinations and a sense of anxiety has led to the recommendation that, in clinical use, *S*-(+)-ketamine should always be combined with a hypnotic drug or sedative [35].

## 2 Clinical applications in children

### 2.1 Premedication

Psychological distress and preoperative fear have been reported in up to 60% of the children, especially in those aged 1–5 years [36,37,38,39]. Furthermore, it was demonstrated that anesthesia is the most frightening aspect of the hospital stay by children [40]. Unfortunately, anesthetic premedication is not a standard practice. As a further issue, children who receive intravenous premedication always refer to the event as a painful experience and one of the most common sources of perioperative pain [41]. Conversely, intranasal, oral, or endorectal drug administration is easily accomplished with minimal discomfort. Furthermore, there is no risk of needle injury, and no special technical skills are required.

The most common agent used for pre-anesthesia in children is the benzodiazepine midazolam. However, there are concerns regarding its ability to prevent emergence delirium (ED) or to cause adequate anxiolysis in children [42,43]. Ketamine, alone or in combination with midazolam, has gained popularity also for premedication purposes [44,45,46]. When orally administered, ketamine (4–6 mg/kg) combined with atropine (0.02 mg/kg) and midazolam (0.1–0.5 mg/kg, up to 10–15 mg) can produce deep sedation [47]. A dose of intramuscular ketamine (2–4 mg/kg), combined with atropine (0.02 mg/kg) and midazolam (0.05 mg/kg), is generally reserved for children who refuse oral premedication or those in which lighter premedication has previously failed. Higher doses of intramuscular ketamine (up to 10 mg/kg), in combination with atropine and midazolam, can be administered to children with difficult venous access or when venous access is required for induction to provide better conditions for the insertion of a venous catheter [48].

### 2.2 Placement of the venous access in uncooperative children

Children commonly experience anxiety and fear during venipuncture [49]. The emotionally traumatic event experienced during medical care can determine or reinforce a child’s negative view of medical personnel, and this can negatively affect any future care needs [50]. Consequently, it is mandatory to address such experiences. Among the various nonpharmacological measures proposed, sedation is the option commonly used to facilitate this process. Among other drugs, ketamine plays a key role in the sedation of children undergoing various procedures in different clinical settings [43,51].

Even more, an uncooperative child represents a special challenge; in this clinical scenario, the attempt to find venous access would represent a difficult task to be accomplished. In fact, the sight of a needle often causes considerable anxiety and fear, and the trauma associated with needle puncture, especially in cases of repeated attempts or clinical history with previous episodes of venipuncture, can further enhance these anxious states with consequent possible harm to the child or the healthcare staff involved. In the case of the uncooperative and combative child, the risk associated with this procedure would be amplified.

The choice of sedative may depend on the professional preferences of the operator, the local context, and the practices commonly used within each facility. However, the pharmacological properties of ketamine make it an advantageous drug for the sedation required for this procedure [25]. Administered orally or intramuscularly in combination with other drugs such as midazolam or in a cocktail to be taken orally “masked” with glucose solution or fruity syrups, it has been shown to provide adequate procedural sedation in various clinical contexts including emergency room, pediatric cardiac catheterization laboratory, and the preoperative area [47,48]. Uncooperative and oppositional children may be more susceptible to a pleasant oral route or rapid intramuscular injection. These routes may produce a more gradual sedation with fewer psychological sequelae. Intramuscular ketamine sedation also provides a rapid onset of action, which can be of paramount importance when rapid vascular access is needed. Furthermore, its ability to preserve the airways and sympathomimetic properties favor its use in emergency situations. In these contexts, the risk of inhalation is high, and hemodynamic instability precludes the use of other sedatives, which can compromise the hemodynamics and pose a risk of airway obstruction or apnea [1].

### 2.3 Rapid sequence induction

Rapid sequence intubation (RSI) is a well-known method of anesthesia induction performed generally in emergency setting with the objective of preventing pulmonary aspiration of gastric contents [52]. Together with a fast-acting neuromuscular blocking agent, the ideal induction agent to facilitate endotracheal intubation is represented by a drug with fast-acting and short duration of effect. In the emergency setting, patients are at high risk of aspiration due to altered conscious levels, resulting in impaired laryngeal reflexes and inadequate starvation periods. Delayed gastric emptying, incompetent lower esophageal sphincter, hiatus hernia, pregnancy, and neuromuscular and metabolic disease represent other risk factors for aspiration [53]. Consequently, RSI plays a crucial role in airway management in the emergency setting. In this scenario, a serious safety concern is the risk of severe hypotension. The hemodynamic control is particularly important for patients in shock. According to the pathophysiology of the shock, the child responds to hypovolemia with an increase in heart rate, and if this compensation fails, hypovolemic shock may occur. Thus, because of its limited effects on hemodynamic function, ketamine has become increasingly popular as an induction agent for RSI [54].

Taken together, ketamine fits the profile of an ideal sedative for RSI: rapid onset of action, short duration of action, effective and reliable in producing adequate sedation, and maintaining laryngeal reflexes, with respiratory and cardiovascular stabilities.

Practically, when ketamine is administered intravenously (1–2 mg/kg), dissociative anesthesia is achieved in 1 or 2 min without suppressing the reticular activation system [1]. The patient experiences sedation, analgesia, and amnesia while maintaining normal respiratory function and protective airway reflexes.

Specific concerns about ketamine include the possible increase in intracranial pressure (ICP), increased intraocular pressure (IOP), paradoxical myocardial depression, and psychotomimetic effects. Nevertheless, the traditional concerns about the relation between ketamine and increased ICP and IOP were questioned by more recent evidence that do not show a significant increase in the ICP level following the infusion of ketamine in the context of head injury [55,56,57,58,59]. Filanovsky et al. [60] defined as a “medical myth” ketamine-induced ICP increment. Even more, in a prospective study of 30 children with increased ICP, Bar-Joseph et al. [61] showed that intravenous bolus doses of ketamine (1–1.5 mg/kg) reduced ICP while maintaining stability in mean arterial pressure and cerebral perfusion pressure (CPP). It is important to highlight that, in patients with head injuries, any episode of hypoxia or hypotension can contribute to secondary injuries. Consequently, avoiding hypotension from RSI induced by anesthesia is a more realistic concern than hypothetical problems with the increased ICP.

### 2.4 Difficult airways

Managing difficult airways in the pediatric population can often be a challenge for the anesthetist. Unlike most adult patients, children are uncooperative and intolerant to noxious stimuli. Therefore, in these circumstances, it is advisable to prepare an adequate analgo-sedation plan. Furthermore, informing the anesthetic and surgical team in advance and planning the conduct with any rescue approaches and all the material readily available can guarantee a faster response by the surgical team in case a surgical approach to the airway is necessary. These measures can reduce the time to hypoxia if airway control is not guaranteed.

Ketamine has been shown to be useful in some pediatric patients with difficult airways, both congenital and acquired [62,63]. Combined with dexmedetomidine, ketamine has been shown to be useful as a sedative for fiber-assisted intubation in a child with Treacher Collins syndrome with severe developmental delay [62]. Similarly, children with other difficult airway syndromes, such as Pierre Robin and Goldenhar, may benefit from using ketamine in addition to other anesthetics while securing the airway [64]. The risk of causing airway obstruction or apnea in patients with these problems, always difficult to ventilate in a face mask and intubate, precludes the use of other intravenous (e.g., propofol) or inhaled (e.g., sevoflurane) anesthetics as induction agents because they can increase the risk of apnea. In fact, the level of anesthesia necessary for the child to tolerate fiber-assisted intubation can often cause apnea and/or airway obstruction, particularly in the case of propofol [65]. Apnea and airway obstruction in a syndromic child with a potentially unmanageable airway wearing a face mask and cannot be intubated can lead to catastrophic consequences. In these circumstances, the combination of ketamine and dexmedetomidine may be indicated as it preserves respiratory function, keeps the airways open, and provides adequate analgo-sedation to allow successful fibro-assisted intubation [62,66]. Furthermore, this drug combination shows some mutually complementary qualities. The sympathomimetic qualities of ketamine can be counterbalanced by the sympatholytic effects of dexmedetomidine. Conversely, the excess secretions caused by ketamine can be alleviated by the xerostomia effect of dexmedetomidine. Even more, since ketamine does not depress cough and swallow reflexes, usage of local anesthetics, such as a lidocaine spray, may be indicated to decrease reactivity during the procedure [67].

### 2.5 Reactivity of the upper airways

In pediatric anesthesia, upper respiratory tract infections (URIs) and asthma are common problems. In these patients, during the perioperative period, the high airway reactivity predisposes to the development of a range of respiratory complications including laryngospasm, bronchospasm, and episodes of desaturation [68]. Case referral is common when a patient presented with signs and symptoms of URIs or asthma for the increased risk of perioperative pulmonary complications. However, evidence has supported the use of an individualized risk assessment [69,70,71]. A recent URI, a history of airway hyperactivity, and the presence of active nasal congestion with secretions predispose the patient to a greater likelihood of developing complications [72]. Moreover, asthma can also lead to a life-threatening situation for a patient who is a candidate for general anesthesia. Like URI, asthma can sensitize the airways to any stimulus and is associated with a significant increase in the production of airway secretions. Indeed, the incidence of bronchospasm in asthmatics under general anesthesia has been reported between 0.17 and 4.2% [73]. Furthermore, the complications of the disease vary widely among patients, which requires careful individual risk assessment before any elective regimen anesthesia. Factors such as the use of bronchodilator medications and a recent symptomatic exacerbation, particularly those requiring medical attention, should be of concern during preoperative evaluation and may also require further medical evaluation before proceeding [74].

Prevention of the production of secretions and other causes of airway stimulation are key components in the anesthetic management of patients with a history of URI and asthma. Typical measures include aspiration of secretions under deep anesthesia, using a laryngeal mask as an adequate alternative to the endotracheal tube, preferring the face mask when possible, and ensuring adequate depth of anesthesia before any manipulation of the airways [75,76]. Premedication with anticholinergics and bronchodilators to reduce secretions and prevent bronchospasm are additional precautionary measures to consider [72].

Ketamine may play an important role in the induction and maintenance of anesthesia in patients considered at risk for active airway disease, especially in emergency situations when anesthesia cannot be postponed. Indeed, its usefulness in asthmatics has been recognized for decades; its bronchodilator effects can counteract the risk of bronchospasm induced by airway stimulation [75].

In an early study, the authors demonstrated that this effect is primarily mediated by ketamine-induced inhibition of neuro-mediated bronchoconstriction [76]. Furthermore, in the perioperative period, preservation of respiratory and lung function can reduce the incidence of desaturation and obviate the need for mechanical ventilation and endotracheal intubation, the latter being recognized as a risk factor for respiratory complications in patients with a URI or asthma [77,78]. Furthermore, given the risk of increased secretion production with ketamine, which is undesirable in these cases, premedication with anticholinergics may be a useful countermeasure.

### 2.6 General anesthesia in patients with special needs

Ketamine can represent a valid choice as a general anesthetic agent also in particular categories of patients [79,80]. It is indicated during prehospital induction of anesthesia, induction of anesthesia in potentially hemodynamic unstable patients, and induction of anesthesia in patients at risk of bronchospasm.

Even more, ketamine can be indicated in patients with neuromuscular disease at risk of malignant hyperthermia and for the induction of anesthesia in potentially cardiac unstable patients [81]. Children with neuromuscular disorders, potentially at risk of developing malignant hyperthermia, can receive ketamine for general anesthesia without increasing the clinical risk. In such cases, the use of ketamine can prevent the use of volatile anesthetic agents and neuromuscular blockers that can trigger malignant hyperthermia. Ramchandra et al. [82] used ketamine for general anesthesia in children (aged 3 months to 12 years) with floppy infant syndrome and who required diagnostic muscle biopsy. In children who received both intramuscular and intravenous ketamine, adequate anesthesia was obtained, suggesting that ketamine may be considered a safe and effective agent for children with neuromuscular disorders.

Ketamine can also be used for induction of anesthesia in children with cyanogenic heart disease, without worsening left-to-right shunt. Tuğrul et al. [83] reported excellent hemodynamic stability when ketamine was used for maintenance of anesthesia in children (aged 3 months to 12 years) undergoing cardiac surgery for the correction of Fallot’s tetralogy.

### 2.7 Procedural sedation and Non-Operating Room Anesthesia

Over the past decade, the number of procedures performed on children outside the operating room has increased substantially [84,85]. Depending on the nature of the examination, procedure, or treatment, various levels of sedation will be required (from mild to deep up to general anesthesia). An ideal “sedation plan” should eliminate fear and anxiety, gain the child’s cooperation, achieve immobilization to the degree necessary for the planned procedure or treatment, reduce awareness, induce amnesia, decrease pain, and maintain safety.

Nowadays, several pharmacological agents are available for the Pediatric Non-Operating Room Anesthesia (NORA) for pediatric patients (i.e., midazolam, fentanyl, ketamine, dexmedetomidine, etomidate, propofol). An optimal drug for NORA presents the following characteristic: rapid onset, short duration of action, no active metabolites, minimal respiratory and cardiovascular side effects optimal hypnotic, and pain control with minimal risk of delirium. Consequently, the growing trend toward more outpatient surgery outside the operating room has generated interest in ketamine for its rapid action, short duration, safety profile, possible administration by almost any route, and sedative/analgesic effects [86]. Pediatric sedation techniques involving the use of ketamine are therefore developing rapidly. Ketamine has always been largely used in remote areas, disaster situations, and limited recourses due to its effectiveness at low cost [87]. Nowadays, ketamine has become a popular drug also during dentistry, emergency, dermatology, and for interventional radiology procedures in children [88,89]. Ketamine has also been used successfully in awake, uncooperative children with blunt trauma for procedural sedation and analgesia [86].

Ketamine can be used in combination with other sedatives. The association with propofol, called “Ketofol,” is increasingly used, especially in emergency [90,91]. The most described and used mixture is made up of 0.5 mg/kg ketamine and 3 mg/kg plus propofol. Since ketamine and propofol present opposing properties, the association of these two drugs is favorable: less hemodynamic instability, less vomiting, and recovery agitation. Ketamine can also be combined with short-acting benzodiazepines (i.e., 0.05 mg/kg midazolam up to 0.1 mg/kg) or other sedatives; however, if the combination of these drugs is capable of decreasing the risk of emergency phenomena is still debatable [11,92]. Even more, ketamine can be administered in combination with atropine (0.01 mg/kg) to diminish hypersalivation.

Besides intravenous administration of ketamine, intranasal administration, using a mucosal atomization, represents a practical option, especially in uncooperative patients or with the difficult placement of the venous access (e.g., burn patient).

### 2.8 Locoregional anesthesia

In children, regional anesthesia provides excellent intraoperative and postoperative pain control. It can also positively modulate the surgical neuro-endocrine stress response and can facilitate early extubation after neonatal surgery [93,94,95]. There is a broad consensus among pediatric anesthetists that the use of different regional anesthesia techniques is extremely useful in the context of pediatric surgery [85].

Despite the increasing use of peripheral nerve blocking techniques, especially in children older than 3 years, central blocks are still popular and are frequently used in the pediatric setting [96]. Therefore, the cornerstones of pediatric regional anesthesia are still caudal and epidural blocks [97]. Nevertheless, the choice to perform a nerve block implies the goal of obtaining the best possible effect for the patient. Consequently, the use of continuous blocking techniques can represent an optimal choice to extend the effect of the blockade as much as possible in the postoperative period. Even more, it has become popular to use a mixture of different drugs, in addition to local anesthetic, to achieve prolonged beneficial effects of the block [98].

Evidence is increased regarding peripheral nerve blocks and the additional use of ketamine and α2-agonists [99,100,101]. As regard neuraxial blocks, the matter is substantially more complex. A quantitative systematic review of randomized controlled trials on the efficacy and safety of ketamine used for caudal block showed that ketamine in addition to local anesthetic prolonged postoperative pain relief with minimum adverse effects compared with local anesthetic alone [102]. Also, the coadministration of ketamine and clonidine for caudal block in pediatrics has been described to provide nearly 24 h of effective analgesia after inguinal hernioplasty [103]. However, the fear of possible neurotoxical effects after the administration of ketamine for neuraxial application has led to a limited use of ketamine for neuraxial block. The current problem related to exposure to anesthetic agents at an early age has made it necessary to test for any potential neurodegenerative effects [104]. It is advisable that drugs used as adjuvants in nerve blocks, in particular in neuraxial blocks, have an appropriate safety profile. The drugs must be free of excipients as various preservatives are known to possess neurotoxic properties. It is also important to evaluate the possible toxic role of repeated doses of ketamine in relation to children’s age. The animal model showed that in doses commonly used for regional anesthesia, ketamine did not show neurotoxic effect [105,106]. However, the concern regarding the safety profile of these adjuvants due to their potential neurotoxicity and neurological complications necessitates a deeper understanding.

### 2.9 Analgesia

Ketamine presents an important analgesic action. In addition to its role as an NMDA receptor antagonist, ketamine induces an analgesic effect by inhibiting nitric oxide synthase and via opioid receptors [24,107]. Over the past decade, renewed interest has centered on the use of ketamine as a co-analgesic infusion for intraoperative and postoperative pain, tumor-related pain, or neuropathic pain [108]. Consequently, ketamine can be used for the management of acute or chronic pain and for the prevention of pain chronification [22]. Even more, ketamine presents an opioid-sparing effect, and it is useful in the reduction of hyperalgesia phenomenon.

Ketamine is widely used as an adjunct to other analgesics during the perioperative period. Studies report the potentiation of opioid-induced analgesia and the opioid-sparing effect of ketamine. Tucker et al. [109] investigated the dose of ketamine that can provide clinical benefit through co-administration with opioids. A serum concentration of ketamine around 30–120 ng/mL can potentiate the antinociceptive effect of fentanyl.

Ketamine can be used for the prevention and treatment of perioperative pain and pain not related to surgery, especially in combination with other analgesic drugs. Continuous subcutaneous injections of ketamine (0.1 mg/kg/h) provided sufficient analgesia with minimal adverse events in trauma patients [110]. Continuous infusion of low-dose ketamine and patient-controlled analgesia (PCA) with morphine for postoperative analgesia in a 13-year-old girl undergoing scoliosis correction was also reported [111]. White and Karsli [112] reported the use of a long-term infusion of ketamine, as an opioid adjuvant, for 37 days in a 9-year-old boy with a 42% body surface burn. The authors reported that ketamine provided excellent analgesia and was well tolerated. Becke et al. [113] administered low-dose intraoperative ketamine to reduce postoperative pain and morphine consumption in infants and children undergoing urological surgery. Ketamine has also been effectively used intravenously and via peritonsillar infiltration to reduce postoperative pain after adenotonsillectomy [114,115]. The combination of intranasal sufentanil and midazolam was compared with intranasal ketamine and midazolam for sedation and postoperative analgesia in children undergoing tooth extractions [116]. Children in the sufentanil group received 1 μg/kg sufentanil and 0.3 mg/kg midazolam 20 min before induction of anesthesia. Children of the ketamine group received 5 mg/kg ketamine and 0.3 mg/kg midazolam intranasally. In both groups, effective postoperative analgesia was provided for multiple tooth extractions; in the sufentanil group, 72% of the children did not need rescue analgesia, compared with 52% in the ketamine group.

Finally, there are numerous reports of the use of ketamine for the management of cancer-related pain [117,118]. In a 2-year-old child with severe cancer pain, Tsui et al. [119] demonstrated the effective analgesic properties of a ketamine infusion used in combination with morphine. Furthermore, several studies showed the efficacy of continuous infusion of low-dose ketamine for the control of neuropathic pain [120,121].

### 2.10 Prevention of emergency delirium

This complication is defined as “perceptual disturbances and psychomotor agitation that most commonly occurs in preschool children in the early post anesthetic period” [122,123]. Emergence from general anesthesia is often uneventful; however, delirium may arise during awakening from anesthesia, with a variable incidence ranging from 10 to 80% [124,125]. This variability in reported rates is likely due to a lack of real understanding of ED and definition variations [126]. Episodes of ED are usually short-lived; however, they increase the risk of self-harm and delayed discharge, require additional nursing care, and may increase medical care costs [127]. Furthermore, the long-term psychological implications of ED have not been thoroughly evaluated; there may be an association between ED and certain behaviors such as eating disorders, sleep disturbances, and separation anxiety [38]. Recent literature discusses the cause-and-effect relationship between ED and pain, suggesting that they often occur simultaneously but are sometimes independent.

Ketamine has been used in several studies with various dosages and methods of administration for the prevention of postoperative delirium [46,128]. When administered nasally in children premedicated with oral midazolam, it causes a significant reduction in ED (3.8%) compared to the group administered with alfentanil (36%) or with placebo (40.7%). Compared to propofol (1 mg/kg) or placebo after sevoflurane induction in patients premedicated with oral midazolam, an intravenous bolus of ketamine (0.25 mg/kg) reduced ED by 15% [129]. The ketamine group also presented symptoms of ED but with severe insensitivity than the propofol and placebo groups. In another study in patients undergoing adenotonsillectomy, an intraoperative bolus of low-dose ketamine (0.15 mg/kg) followed by dexmedetomidine (0.3 μg/kg) before the end of surgery under anesthesia with sevoflurane decreased the incidence and severity of ED compared to that experienced by a control group that received placebo (11 vs 47%) [130]. Two studies examining the administration of 0.25 mg/kg ketamine as a bolus at the end of a procedure (tonsillectomy/adenoidectomy) compared to placebo showed a reduced risk of ED [131,132]. The incidence of ED was also lower in the groups that received 0.25 or 0.5 mg/kg ketamine compared to the control group. However, there was no significant difference in the incidence of ED between the two groups [132]. Abu-Shahwan and Chowdary [131] conducted a double-blind study involving 85 patients undergoing dental repair. The authors found that ED occurred in 16.6% of the children in the ketamine group in comparison to 34.2% in the placebo group. A study evaluating a propofol-based ED prevention strategy for repetitive radiotherapy sessions in children reported that adding low-dose ketamine to propofol infusion in a 1:10 ratio may reduce incidence by ED [133]. One study used a nonopioid approach to manage pain and reduce the incidence of ED in children undergoing adenotonsillectomy and found that oral premedication with dextromethorphan (1 mg/kg) or ketamine (5 mg/kg) significantly reduced the incidence of sevoflurane-related postoperative ED with no reported side effects [134].

### 2.11 Pediatric intensive care

In pediatric intensive care units (PICUs), adequate sedation of mechanically ventilated children is critical to ensure patient comfort and safety, to improve adaptation to mechanical ventilation, and to prevent accidental extubation [135,136].

Although opioids and benzodiazepines are often used as first-choice drug for analgosedation in PICUs, numerous adverse effects including hemodynamic instability, decreased gastrointestinal motility, delirium, and iatrogenic withdrawal symptoms are observed. Furthermore, tolerance develops rapidly in many children, and careful de-escalation of drugs must be planned to maintain an effective level of sedation and avoid abstinence during drug de-escalation. Therefore, while opioids and benzodiazepines remain the mainstay of sedative agents used in PICUs, effective alternatives are warranted. Despite propofol infusion is commonly used in sedative intensive care regimens for adults, it exposes children to a high risk of severe metabolic acidosis and cardiovascular collapse, the so-called propofol infusion syndrome [137]. In the last year, dexmedetomidine has increasingly been used for PICU sedation and, although this α2 agonist has the distinct advantage of minimal respiratory depression, its side effect profile may include hypotension and bradycardia, which may limit its use in some patients [138]. Ketamine is a fast-acting general anesthetic with analgesic and sedative activity that represents an additional option for sedation in PICU patients. Moreover, among other properties, ketamine maintains lung compliance by reducing airway resistance and has been shown to be beneficial in children with asthma [139]. Unlike other medications used in PICU sedation, ketamine has mainly beneficial effects on the cardiovascular system. Due to its favorable profile on hemodynamic and respiratory functions, ketamine has been used for sedation of mechanically ventilated patients [140,141,142]. On these bases, Tobias et al. [143] first described the use of continuous ketamine infusion to successfully provide sedation and analgesia to five seriously ill children. Later, Hartvig et al. [144] reported a study of 10 children who received continuous ketamine infusion after heart surgery with effective analgesia and sedation.

The optimal dosage of ketamine in continuous infusion for the sedation of children in PICU ranges from 0.2 to 6 mg/kg/h in relation to the goal to be achieved (i.e., sedation, prevention of tolerance to opioids and benzodiazepines, pharmacological weaning) and to the various phases of the planned clinical path [142,145]. Even more, in the PICU, ketamine can be used as a part of a multimodal pharmacological approach to reduce the dose of short-acting benzodiazepine and opioids with the aim of reducing the side effects of these drugs. It can be concluded that ketamine certainly represents an alternative agent for pediatric patients requiring sedation and analgesia during mechanical ventilation, but further randomized studies are needed to better define its role [135].

## 3 Conclusions

Ketamine represents a fundamental drug for anesthetists and intensive care practitioners. Besides its leading role in prehospital and emergency settings in respiratory or cardiovascular unstable patients, this drug can also be used as an adjuvant to prolong the duration and potentiate the effect of a single medication. However, a very important concern is the theoretical risk of dose-dependent neurodegenerative effect for infants and young children. This aspect is worth to evaluate deeply in further *in vivo* and *in vitro* trials. However, ketamine still represents a drug with a lot of potential in the practice of pediatric anesthesia.

## References

[1] Cromhout A. Ketamine: its use in the emergency department. Emerg Med (Fremantle). 2003;15(2):155–9. 10.1046/j.1442-2026.2003.00433.x 12675625

[2] Kurdi MS, Theerth KA, Deva RS. Ketamine: Current applications in anesthesia, pain, and critical care. Anesth Essays Res. 2014;8(3):283–90. 10.4103/0259-1162.143110 PMC4258981 25886322

[3] Bergman SA. Ketamine: review of its pharmacology and its use in pediatric anesthesia. Anesth Prog. 1999;46(1):10–20. PMC2148883 10551055

[4] Canet J, Castillo J. Ketamine: a familiar drug we trust. Anesthesiology. 2012;116(1):6–8. 10.1097/ALN.0b013e31823da398 22080003

[5] von Ungern-Sternberg BS, Regli A, Frei FJ, Ritz EM, Hammer J, Schibler A, et al. A deeper level of ketamine anesthesia does not affect functional residual capacity and ventilation distribution in healthy preschool children. Paediatr Anaesth. 2007;17(12):1150–5. 10.1111/j.1460-9592.2007.02335.x 17986033

[6] Zanos P, Moaddel R, Morris PJ, Riggs LM, Highland JN, Georgiou P, et al. Ketamine and Ketamine Metabolite Pharmacology: Insights into Therapeutic Mechanisms. Pharmacol Rev. 2018;70(3):621–60. 10.1124/pr.117.015198 PMC6020109 29945898

[7] White JM, Ryan CF. Pharmacological properties of ketamine. Drug Alcohol Rev. 1996;15(2):145–55. 10.1080/09595239600185801 16203365

[8] Green SM, Krauss B. Clinical practice guideline for emergency department ketamine dissociative sedation in children. Ann Emerg Med. 2004;44(5):460–71. 10.1016/S0196064404006365 15520705

[9] Green SM, Roback MG, Krauss B, Brown L, McGlone RG, Agrawal D, et al. Predictors of emesis and recovery agitation with emergency department ketamine sedation: an individual-patient data meta-analysis of 8,282 children. Ann Emerg Med. 2009;54(2):171–80. 10.1016/j.annemergmed.2009.04.004 19501426

[10] Sener S, Eken C, Schultz CH, Serinken M, Ozsarac M. Ketamine with and without midazolam for emergency department sedation in adults: a randomized controlled trial. Ann Emerg Med. 2011;57(2):109–14. 10.1016/j.annemergmed.2010.09.010 20970888

[11] Wathen JE, Roback MG, Mackenzie T, Bothner JP. Does midazolam alter the clinical effects of intravenous ketamine sedation in children? A double-blind, randomized, controlled, emergency department trial. Ann Emerg Med. 2000;36(6):579–88. 10.1067/mem.2000.111131 11097698

[12] Chudnofsky CR, Weber JE, Stoyanoff PJ, Colone PD, Wilkerson MD, Hallinen DL, et al. A combination of midazolam and ketamine for procedural sedation and analgesia in adult emergency department patients. Acad Emerg Med. 2000;7(3):228–35. 10.1111/j.1553-2712.2000.tb01064.x 10730829

[13] Perumal DK, Adhimoolam M, Selvaraj N, Lazarus SP, Mohammed MAR. Midazolam premedication for Ketamine-induced emergence phenomenon: A prospective observational study. J Res Pharm Pract. 2015;4(2):89–93. 10.4103/2279-042X.155758 PMC4418142 25984547

[14] Pennybaker SJ, Luckenbaugh DA, Park LT, Marquardt CA, Zarate CA, Jr. Ketamine and Psychosis History: Antidepressant Efficacy and Psychotomimetic Effects Postinfusion. Biol Psychiatry. 2017;82(5):e35–e6. 10.1016/j.biopsych.2016.08.041 PMC5859935 28262250

[15] da Frota Ribeiro CM, Sanacora G, Hoffman R, Ostroff R. The Use of Ketamine for the Treatment of Depression in the Context of Psychotic Symptoms: To the Editor. Biol Psychiatry. 2016;79(9):e65–e6. 10.1016/j.biopsych.2015.05.016 26212896

[16] Choudhury D, Autry AE, Tolias KF, Krishnan V. Ketamine: neuroprotective or neurotoxic? Front Neurosci. 2021;15:672526. 10.3389/fnins.2021.672526 PMC8461018 34566558

[17] Mellon RD, Simone AF, Rappaport BA. Use of anesthetic agents in neonates and young children. Anesth Analg. 2007;104(3):509–20. 10.1213/01.ane.0000255729.96438.b0 17312200

[18] Li X, Li Y, Zhao J, Li L, Wang Y, Zhang Y, et al. Administration of ketamine causes autophagy and apoptosis in the rat fetal hippocampus and in PC12 Cells. Front Cell Neurosci. 2018;12:21. 10.3389/fncel.2018.00021 PMC5801406 29456493

[19] Seecheran R, Narayansingh R, Giddings S, Rampaul M, Furlonge K, Abdool K, et al. Atrial arrhythmias in a patient presenting with Coronavirus Disease-2019 (COVID-19) Infection. J Investig Med High Impact Case Rep. 2020;8:2324709620925571. 10.1177/2324709620925571 PMC7218462 32370558

[20] Davidson A, McCann ME, Morton N. Anesthesia neurotoxicity in neonates: the need for clinical research. Anesth Analg. 2007;105(3):881–2. 10.1213/01.ane.0000269692.57331.48 17717261

[21] Loepke AW, Soriano SG. An assessment of the effects of general anesthetics on developing brain structure and neurocognitive function. Anesth Analg. 2008;106(6):1681–707. 10.1213/ane.0b013e318167ad77 18499597

[22] Trimmel H, Helbok R, Staudinger T, Jaksch W, Messerer B, Schöchl H, et al. S(+)-ketamine: Current trends in emergency and intensive care medicine. Wien Klin Wochenschr. 2018;130(9–10):356–66. 10.1007/s00508-017-1299-3 PMC6061669 29322377

[23] Scheppke KA, Braghiroli J, Shalaby M, Chait R. Prehospital Use of IM Ketamine for Sedation of Violent and Agitated Patients. West J Emerg Med. 2014;15(7):736–41. 10.5811/westjem.2014.9.23229 PMC4251212 25493111

[24] Gao M, Rejaei D, Liu H. Ketamine use in current clinical practice. Acta Pharmacol Sin. 2016;37(7):865–72. 10.1038/aps.2016.5 PMC4933765 27018176

[25] Marland S, Ellerton J, Andolfatto G, Strapazzon G, Thomassen O, Brandner B, et al. Ketamine: use in anesthesia. CNS Neurosci Ther. 2013;19(6):381–9. 10.1111/cns.12072 PMC6493613 23521979

[26] Grijalva J, Vakili K. Neonatal liver physiology. Semin Pediatr Surg. 2013;22(4):185–9. 10.1053/j.sempedsurg.2013.10.006 24331092

[27] Piñeiro-Carrero VM, Piñeiro EO. Liver. Pediatrics. 2004;113(4 Suppl):1097–106. 15060205

[28] Vaughns JD, Conklin LS, Long Y, Zheng P, Faruque F, Green DJ, et al. Obesity and Pediatric Drug Development. J Clin Pharmacol. 2018;58(5):650–61. 10.1002/jcph.1054 PMC7335432 29350758

[29] Domino EF. Taming the ketamine tiger. 1965. Anesthesiology. 2010;113(3):678–84. 10.1097/ALN.0b013e3181ed09a2 20693870

[30] Mion G, Villevieille T. Ketamine pharmacology: an update (pharmacodynamics and molecular aspects, recent findings). CNS Neurosci Ther. 2013;19(6):370–80. 10.1111/cns.12099 PMC6493357 23575437

[31] Doenicke A, Kugler J, Mayer M, Angster R, Hoffmann P. Ketamine racemate or S-(+)-ketamine and midazolam. The effect on vigilance, efficacy and subjective findings. Anaesthesist. 1992;41(10):610–8. 1443509

[32] White PF, Way WL, Trevor AJ. Ketamine--its pharmacology and therapeutic uses. Anesthesiology. 1982;56(2):119–36. 10.1097/00000542-198202000-00007 6892475

[33] Adams HA, Werner C. From the racemate to the eutomer: (S)-ketamine. Renaissance of a substance?. Anaesthesist. 1997;46(12):1026–42. 10.1007/s001010050503 9451486

[34] Engelhardt W. Recovery and psychomimetic reactions following S-(+)-ketamine. Anaesthesist. 1997;46(Suppl 1):S38–42. 10.1007/pl00002463 9163277

[35] Mathisen LC, Skjelbred P, Skoglund LA, Øye I. Effect of ketamine, an NMDA receptor inhibitor, in acute and chronic orofacial pain. Pain. 1995;61(2):215–20. 10.1016/0304-3959(94)00170-J 7659431

[36] Vernon DT, Foley JM, Sipowicz RR, Schulman JL. The psychological responses of children to hospitalization and illness. Kango Kenkyu. 1971;4(1):45–54. 5313472

[37] Melamed BG, Siegel LJ. Reduction of anxiety in children facing hospitalization and surgery by use of filmed modeling. J Consult Clin Psychol. 1975;43(4):511–21. 10.1037/h0076896 1159149

[38] Kain ZN, Mayes LC, O’Connor TZ, Cicchetti DV. Preoperative anxiety in children. Predictors and outcomes. Arch Pediatr Adolesc Med. 1996;150(12):1238–45. 10.1001/archpedi.1996.02170370016002 8953995

[39] Humphrey GB, Boon CM, van Linden van den Heuvell GF, van de Wiel HB. The occurrence of high levels of acute behavioral distress in children and adolescents undergoing routine venipunctures. Pediatrics. 1992;90(1 Pt 1):87–91. 1614786

[40] Corman HH, Hornick EJ, Kritchman M, Terestman N. Emotional reactions of surgical patients to hospitalization, anesthesia and surgery. Am J Surg. 1958;96(5):646–53. 10.1016/0002-9610(58)90466-5 13583329

[41] Bisogni S, Dini C, Olivini N, Ciofi D, Giusti F, Caprilli S, et al. Perception of venipuncture pain in children suffering from chronic diseases. BMC Res Notes. 2014;7:735. 10.1186/1756-0500-7-735 PMC4210598 25326685

[42] Köner O, Türe H, Mercan A, Menda F, Sözübir S. Effects of hydroxyzine-midazolam premedication on sevoflurane-induced paediatric emergence agitation: a prospective randomised clinical trial. Eur J Anaesthesiol. 2011;28(9):640–5. 10.1097/EJA.0b013e328344db1a 21822077

[43] Neville DN, Hayes KR, Ivan Y, McDowell ER, Pitetti RD. Double-blind Randomized Controlled Trial of Intranasal Dexmedetomidine Versus Intranasal Midazolam as Anxiolysis Prior to Pediatric Laceration Repair in the Emergency Department. Acad Emerg Med. 2016;23(8):910–7. 10.1111/acem.12998 27129606

[44] Oyedepo O, Nasir A, Abdur-Rahman L, Kolawole I, Bolaji B, Ige O. Efficacy and safety of oral ketamine premedication in children undergoing day case surgery. J West Afr Coll Surg. 2016;6(1):1–15. PMC5342622 28344934

[45] Kim KM, Lee KH, Kim YH, Ko MJ, Jung JW, Kang E. Comparison of effects of intravenous midazolam and ketamine on emergence agitation in children: Randomized controlled trial. J Int Med Res. 2016;44(2):258–66. 10.1177/0300060515621639 PMC5580063 26880794

[46] Bilgen S, Köner Ö, Karacay S, Sancar NK, Kaspar EC, Sözübir S. Effect of ketamine versus alfentanil following midazolam in preventing emergence agitation in children after sevoflurane anaesthesia: a prospective randomized clinical trial. J Int Med Res. 2014;42(6):1262–71. 10.1177/0300060514543039 25217473

[47] Shah S, Apuya J, Gopalakrishnan S, Martin T. Combination of oral ketamine and midazolam as a premedication for a severely autistic and combative patient. J Anesth. 2009;23(1):126–8. 10.1007/s00540-008-0685-4 19234837

[48] Rai K, Hegde AM, Goel K. Sedation in uncooperative children undergoing dental procedures: a comparative evaluation of midazolam, propofol and ketamine. J Clin Pediatr Dent. 2007;32(1):1–4. 10.17796/jcpd.32.1.v74872j8n74qu81k 18274461

[49] Deacon B, Abramowitz J. Fear of needles and vasovagal reactions among phlebotomy patients. J Anxiety Disord. 2006;20(7):946–60. 10.1016/j.janxdis.2006.01.004 16460906

[50] Lerwick JL. Minimizing pediatric healthcare-induced anxiety and trauma. World J Clin Pediatr. 2016;5(2):143–50. 10.5409/wjcp.v5.i2.143 PMC4857227 27170924

[51] Priestley SJ, Taylor J, McAdam CM, Francis P. Ketamine sedation for children in the emergency department. Emerg Med (Fremantle). 2001;13(1):82–90. 10.1046/j.1442-2026.2001.00184.x 11476419

[52] Stewart JC, Bhananker S, Ramaiah R. Rapid-sequence intubation and cricoid pressure. Int J Crit Illn Inj Sci. 2014;4(1):42–9. 10.4103/2229-5151.128012 PMC3982370 24741497

[53] Nason KS. Acute intraoperative pulmonary aspiration. Thorac Surg Clin. 2015;25(3):301–7. 10.1016/j.thorsurg.2015.04.011 PMC4517287 26210926

[54] Sehdev RS, Symmons DA, Kindl K. Ketamine for rapid sequence induction in patients with head injury in the emergency department. Emerg Med Australas. 2006;18(1):37–44. 10.1111/j.1742-6723.2006.00802.x 16454773

[55] Russo E, Santonastaso DP, Gamberini E, Circelli A, Martino C, Agnoletti V. Ketamine in Neurocritical Care. J Intensive Care Med. 2020;885066620912719. 10.1177/0885066620912719 32193979

[56] Mayberg TS, Lam AM, Matta BF, Domino KB, Winn HR. Ketamine does not increase cerebral blood flow velocity or intracranial pressure during isoflurane/nitrous oxide anesthesia in patients undergoing craniotomy. Anesth Analg. 1995;81(1):84–9. 10.1097/00000539-199507000-00017 7598288

[57] Bourgoin A, Albanèse J, Léone M, Sampol-Manos E, Viviand X, Martin C. Effects of sufentanil or ketamine administered in target-controlled infusion on the cerebral hemodynamics of severely brain-injured patients. Crit Care Med. 2005;33(5):1109–13. 10.1097/01.ccm.0000162491.26292.98 15891344

[58] Bourgoin A, Albanèse J, Wereszczynski N, Charbit M, Vialet R, Martin C. Safety of sedation with ketamine in severe head injury patients: comparison with sufentanil. Crit Care Med. 2003;31(3):711–7. 10.1097/01.CCM.0000044505.24727.16 12626974

[59] Schmittner MD, Vajkoczy SL, Horn P, Bertsch T, Quintel M, Vajkoczy P, et al. Effects of fentanyl and S(+)-ketamine on cerebral hemodynamics, gastrointestinal motility, and need of vasopressors in patients with intracranial pathologies: a pilot study. J Neurosurg Anesthesiol. 2007;19(4):257–62. 10.1097/ANA.0b013e31811f3feb 17893578

[60] Filanovsky Y, Miller P, Kao J. Myth: Ketamine should not be used as an induction agent for intubation in patients with head injury. Cjem. 2010;12(2):154–7. 10.1017/s1481803500012197 20219164

[61] Bar-Joseph G, Guilburd Y, Tamir A, Guilburd JN. Effectiveness of ketamine in decreasing intracranial pressure in children with intracranial hypertension. J Neurosurg Pediatr. 2009;4(1):40–6. 10.3171/2009.1.PEDS08319 19569909

[62] Iravani M, Wald SH. Dexmedetomidine and ketamine for fiberoptic intubation in a child with severe mandibular hypoplasia. J Clin Anesth. 2008;20(6):455–7. 10.1016/j.jclinane.2008.03.012 18929288

[63] Paterson NA. Management of an unusual pediatric difficult airway using ketamine as a sole agent. Paediatr Anaesth. 2008;18(8):785–8. 10.1111/j.1460-9592.2008.02651.x 18613935

[64] Xue FS, Luo MP, Xu YC, Liao X. Airway anesthesia for awake fiberoptic intubation in management of pediatric difficult airways. Paediatr Anaesth. 2008;18(12):1264–5. 10.1111/j.1460-9592.2008.02778.x 19076599

[65] Bahk JH, Sung J, Jang IJ. A comparison of ketamine and lidocaine spray with propofol for the insertion of laryngeal mask airway in children: a double-blinded randomized trial. Anesth Analg. 2002;95(6):1586–9. 10.1097/00000539-200212000-00021 12456421

[66] Sinha SK, Joshiraj B, Chaudhary L, Hayaran N, Kaur M, Jain A. A comparison of dexmedetomidine plus ketamine combination with dexmedetomidine alone for awake fiberoptic nasotracheal intubation: A randomized controlled study. J Anaesthesiol Clin Pharmacol. 2014;30(4):514–9. 10.4103/0970-9185.142846 PMC4234788 25425777

[67] Aroni F, Iacovidou N, Dontas I, Pourzitaki C, Xanthos T. Pharmacological aspects and potential new clinical applications of ketamine: reevaluation of an old drug. J Clin Pharmacol. 2009;49(8):957–64. 10.1177/0091270009337941 19546251

[68] Lawal I, Bakari A. Reactive airway and anaesthesia: challenge to the anaesthetist and the way forward. Afr Health Sci. 2009;9(3):167–9. PMC2887028 20589145

[69] Hallas HW, Chawes BL, Rasmussen MA, Arianto L, Stokholm J, Bønnelykke K, et al. Airway obstruction and bronchial reactivity from age 1 month until 13 years in children with asthma: A prospective birth cohort study. PLoS Med. 2019;16(1):1002722. 10.1371/journal.pmed.1002722 PMC6324782 30620743

[70] Woods BD, Sladen RN. Perioperative considerations for the patient with asthma and bronchospasm. Br J Anaesth. 2009;103(Suppl 1):i57–65. 10.1093/bja/aep271 20007991

[71] Parnis SJ, Barker DS, Van Der Walt JH. Clinical predictors of anaesthetic complications in children with respiratory tract infections. Paediatr Anaesth. 2001;11(1):29–40. 10.1046/j.1460-9592.2001.00607.x 11123728

[72] Tait AR, Malviya S. Anesthesia for the child with an upper respiratory tract infection: still a dilemma? Anesth Analg. 2005;100(1):59–65. 10.1213/01.ANE.0000139653.53618.91 15616052

[73] Bremerich DH. Anesthesia in bronchial asthma. Anasthesiol Intensivmed Notfallmed Schmerzther. 2000;35(9):545–58. 10.1055/s-2000-7091 11050961

[74] Burburan SM, Xisto DG, Rocco PR. Anaesthetic management in asthma. Minerva Anestesiol. 2007;73(6):357–65. 17115010

[75] Corssen G, Gutierrez J, Reves JG, Huber FC, Jr. Ketamine in the anesthetic management of asthmatic patients. Anesth Analg. 1972;51(4):588–96. 5064793

[76] Brown RH, Wagner EM. Mechanisms of bronchoprotection by anesthetic induction agents: propofol versus ketamine. Anesthesiology. 1999;90(3):822–8. 10.1097/00000542-199903000-00025 10078684

[77] Warner DO, Warner MA, Barnes RD, Offord KP, Schroeder DR, Gray DT, et al. Perioperative respiratory complications in patients with asthma. Anesthesiology. 1996;85(3):460–7. 10.1097/00000542-199609000-00003 8853074

[78] Hendaus MA, Jomha FA, Alhammadi AH. Is ketamine a lifesaving agent in childhood acute severe asthma? Ther Clin Risk Manag. 2016;12:273–9. 10.2147/TCRM.S100389 PMC4768891 26955277

[79] Racca F, Mongini T, Wolfler A, Vianello A, Cutrera R, Del Sorbo L, et al. Recommendations for anesthesia and perioperative management of patients with neuromuscular disorders. Minerva Anestesiol. 2013;79(4):419–33. 23419334

[80] Lin C, Durieux ME. Ketamine and kids: an update. Paediatr Anaesth. 2005;15(2):91–7. 10.1111/j.1460-9592.2005.01475.x 15675923

[81] Dershwitz M, Sréter FA, Ryan JF. Ketamine does not trigger malignant hyperthermia in susceptible swine. Anesth Analg. 1989;69(4):501–3. 2782651

[82] Ramchandra DS, Anisya V, Gourie-Devi M. Ketamine monoanaesthesia for diagnostic muscle biopsy in neuromuscular disorders in infancy and childhood: floppy infant syndrome. Can J Anaesth. 1990;37(4 Pt 1):474–6. 10.1007/BF03005630 2340618

[83] Tuğrul M, Camci E, Pembeci K, Telci L, Akpir K. Ketamine infusion versus isoflurane for the maintenance of anesthesia in the prebypass period in children with tetralogy of Fallot. J Cardiothorac Vasc Anesth. 2000;14(5):557–61. 10.1053/jcan.2000.9448 11052438

[84] Gozal D, Drenger B, Levin PD, Kadari A, Gozal Y. A pediatric sedation/anesthesia program with dedicated care by anesthesiologists and nurses for procedures outside the operating room. J Pediatr. 2004;145(1):47–52. 10.1016/j.jpeds.2004.01.044 15238906

[85] Mahmoud M, Mason KP. A forecast of relevant pediatric sedation trends. Curr Opin Anaesthesiol. 2016;29(Suppl 1):S56–67. 10.1097/ACO.0000000000000321 26926335

[86] Bredmose PP, Lockey DJ, Grier G, Watts B, Davies G. Pre-hospital use of ketamine for analgesia and procedural sedation. Emerg Med J. 2009;26(1):62–4. 10.1136/emj.2007.052753 19104109

[87] Mulvey JM, Qadri AA, Maqsood MA. Earthquake injuries and the use of ketamine for surgical procedures: the Kashmir experience. Anaesth Intensive Care. 2006;34(4):489–94. 10.1177/0310057X0603400413 16913348

[88] Green SM, Johnson NE. Ketamine sedation for pediatric procedures: Part 2, Review and implications. Ann Emerg Med. 1990;19(9):1033–46. 10.1016/s0196-0644(05)82569-7 2203290

[89] Zanvettor A, Lederer W, Glodny B, Chemelli AP, Wiedermann FJ. Procedural sedation and analgesia for percutaneous trans-hepatic biliary drainage: Randomized clinical trial for comparison of two different concepts. Open Med (Wars). 2020;15(1):815–21. 10.1515/med-2020-0220 PMC7712221 33336039

[90] Green SM, Andolfatto G, Krauss BS. Ketofol for procedural sedation revisited: pro and con. Ann Emerg Med. 2015;65(5):489–91. 10.1016/j.annemergmed.2014.12.002 25544732

[91] Scheier E, Gadot C, Leiba R, Shavit I. Sedation with the Combination of Ketamine and Propofol in a Pediatric ED: A Retrospective Case Series Analysis. Am J Emerg Med. 2015;33(6):815–7. 10.1016/j.ajem.2015.03.033 25819203

[92] Cevik E, Bilgic S, Kilic E, Cinar O, Hasman H, Acar AY, et al. Comparison of ketamine-low-dose midozolam with midazolam-fentanyl for orthopedic emergencies: a double-blind randomized trial. Am J Emerg Med. 2013;31(1):108–13. 10.1016/j.ajem.2012.06.012 22944555

[93] Wolf AR, Eyres RL, Laussen PC, Edwards J, Stanley IJ, Rowe P, et al. Effect of extradural analgesia on stress responses to abdominal surgery in infants. Br J Anaesth. 1993;70(6):654–60. 10.1093/bja/70.6.654 8392359

[94] Courrèges P, Poddevin F, Lecoutre D, Bayart R. Epidural anesthesia and esophageal atresia. Apropos of a case. Cah Anesthesiol. 1995;43(5):471–4. 8564673

[95] Hodgson RE, Bösenberg AT, Hadley LG. Congenital diaphragmatic hernia repair--impact of delayed surgery and epidural analgesia. S Afr J Surg. 2000;38(2):31–4. 10967692

[96] Ecoffey C, Lacroix F, Giaufré E, Orliaguet G, Courrèges P. Epidemiology and morbidity of regional anesthesia in children: a follow-up one-year prospective survey of the French-Language Society of Paediatric Anaesthesiologists (ADARPEF). Paediatr Anaesth. 2010;20(12):1061–9. 10.1111/j.1460-9592.2010.03448.x 21199114

[97] Lundblad M, Lönnqvist PA. Adjunct analgesic drugs to local anaesthetics for neuroaxial blocks in children. Curr Opin Anaesthesiol. 2016;29(5):626–31. 10.1097/ACO.0000000000000372 27388793

[98] Sanders JC. Paediatric regional anaesthesia, a survey of practice in the United Kingdom. Br J Anaesth. 2002;89(5):707–10. 12393767

[99] Zaman B, Hojjati Ashrafi S, Seyed Siamdoust S, Hassani V, Mohamad Taheri S, Noorizad S. The Effect of Ketamine and Dexamethasone in Combination with Lidocaine on the Onset and Duration of Axillary Block in Hand and Forearm Soft Tissue Surgery. Anesth Pain Med. 2017;7(5):15570. 10.5812/aapm.15570 PMC5903223 29696115

[100] Lönnqvist PA. Alpha-2 adrenoceptor agonists as adjuncts to Peripheral Nerve Blocks in Children--is there a mechanism of action and should we use them? Paediatr Anaesth. 2012;22(5):421–4. 10.1111/j.1460-9592.2012.03821.x 22486904

[101] Lundblad M, Marhofer D, Eksborg S, Lönnqvist PA. Dexmedetomidine as adjunct to ilioinguinal/iliohypogastric nerve blocks for pediatric inguinal hernia repair: an exploratory randomized controlled trial. Paediatr Anaesth. 2015;25(9):897–905. 10.1111/pan.12704 26095747

[102] Schnabel A, Poepping DM, Kranke P, Zahn PK, Pogatzki-Zahn EM. Efficacy and adverse effects of ketamine as an additive for paediatric caudal anaesthesia: a quantitative systematic review of randomized controlled trials. Br J Anaesth. 2011;107(4):601–11. 10.1093/bja/aer258 21846679

[103] Hager H, Marhofer P, Sitzwohl C, Adler L, Kettner S, Semsroth M. Caudal clonidine prolongs analgesia from caudal S(+)-ketamine in children. Anesth Analg. 2002;94(5):1169–72. 10.1097/00000539-200205000-00021 11973182

[104] Walker SM, Yaksh TL. New caudal additives in children: benefit vs. risk? Acta Anaesthesiol Scand. 2009;53(8):1097–8. 10.1111/j.1399-6576.2009.02013.x 19694618

[105] Tobias JD. Caudal epidural block: a review of test dosing and recognition of systemic injection in children. Anesth Analg. 2001;93(5):1156–61. 10.1097/00000539-200111000-00018 11682386

[106] Walker SM, Westin BD, Deumens R, Grafe M, Yaksh TL. Effects of intrathecal ketamine in the neonatal rat: evaluation of apoptosis and long-term functional outcome. Anesthesiology. 2010;113(1):147–59. 10.1097/ALN.0b013e3181dcd71c PMC2904694 20526188

[107] Elia N, Tramèr MR. Ketamine and postoperative pain--a quantitative systematic review of randomised trials. Pain. 2005;113(1–2):61–70. 10.1016/j.pain.2004.09.036 15621365

[108] Grande LA, O’Donnell BR, Fitzgibbon DR, Terman GW. Ultra-low dose ketamine and memantine treatment for pain in an opioid-tolerant oncology patient. Anesth Analg. 2008;107(4):1380–3. 10.1213/ane.0b013e3181733ddd 18806055

[109] Tucker AP, Kim YI, Nadeson R, Goodchild CS. Investigation of the potentiation of the analgesic effects of fentanyl by ketamine in humans: a double-blinded, randomised, placebo controlled, crossover study of experimental pain [ISRCTN83088383]. BMC Anesthesiol. 2005;5(1):2. 10.1186/1471-2253-5-2 PMC1084341 15804361

[110] Gurnani A, Sharma PK, Rautela RS, Bhattacharya A. Analgesia for acute musculoskeletal trauma: low-dose subcutaneous infusion of ketamine. Anaesth Intensive Care. 1996;24(1):32–6. 10.1177/0310057X9602400106 8669651

[111] Tsui BC, Wagner A, Mahood J, Moreau M. Adjunct continuous intravenous ketamine infusion for postoperative pain relief following posterior spinal instrumentation for correction of scoliosis: a case report. Paediatr Anaesth. 2007;17(4):383–6. 10.1111/j.1460-9592.2006.02134.x 17359410

[112] White MC, Karsli C. Long-term use of an intravenous ketamine infusion in a child with significant burns. Paediatr Anaesth. 2007;17(11):1102–4. 10.1111/j.1460-9592.2007.02329.x 17897278

[113] Becke K, Albrecht S, Schmitz B, Rech D, Koppert W, Schüttler J, et al. Intraoperative low-dose S-ketamine has no preventive effects on postoperative pain and morphine consumption after major urological surgery in children. Paediatr Anaesth. 2005;15(6):484–90. 10.1111/j.1460-9592.2005.01476.x 15910349

[114] Dal D, Celebi N, Elvan EG, Celiker V, Aypar U. The efficacy of intravenous or peritonsillar infiltration of ketamine for postoperative pain relief in children following adenotonsillectomy. Paediatr Anaesth. 2007;17(3):263–9. 10.1111/j.1460-9592.2006.02095.x 17263742

[115] Aspinall RL, Mayor A. A prospective randomized controlled study of the efficacy of ketamine for postoperative pain relief in children after adenotonsillectomy. Paediatr Anaesth. 2001;11(3):333–6. 10.1046/j.1460-9592.2001.00676.x 11359593

[116] Roelofse JA, Shipton EA, de la Harpe CJ, Blignaut RJ. Intranasal sufentanil/midazolam versus ketamine/midazolam for analgesia/sedation in the pediatric population prior to undergoing multiple dental extractions under general anesthesia: a prospective, double-blind, randomized comparison. Anesth Prog. 2004;51(4):114–21. PMC2007493 15675259

[117] Sveticic G, Gentilini A, Eichenberger U, Luginbühl M, Curatolo M. Combinations of morphine with ketamine for patient-controlled analgesia: a new optimization method. Anesthesiology. 2003;98(5):1195–205. 10.1097/00000542-200305000-00023 12717142

[118] Klepstad P, Borchgrevink P, Hval B, Flaat S, Kaasa S. Long-term treatment with ketamine in a 12-year-old girl with severe neuropathic pain caused by a cervical spinal tumor. J Pediatr Hematol Oncol. 2001;23(9):616–9. 10.1097/00043426-200112000-00013 11902308

[119] Tsui BC, Davies D, Desai S, Malherbe S. Intravenous ketamine infusion as an adjuvant to morphine in a 2-year-old with severe cancer pain from metastatic neuroblastoma. J Pediatr Hematol Oncol. 2004;26(10):678–80. 10.1097/01.mph.0000140656.96085.2c 27811612

[120] Hocking G, Cousins MJ. Ketamine in chronic pain management: an evidence-based review. Anesth Analg. 2003;97(6):1730–9. 10.1213/01.ANE.0000086618.28845.9B 14633551

[121] Maher DP, Chen L, Mao J. Intravenous Ketamine Infusions for Neuropathic Pain Management: A Promising Therapy in Need of Optimization. Anesth Analg. 2017;124(2):661–74. 10.1213/ANE.0000000000001787 28067704

[122] Banchs RJ, Lerman J. Preoperative anxiety management, emergence delirium, and postoperative behavior. Anesthesiol Clin. 2014;32(1):1–23. 10.1016/j.anclin.2013.10.011 24491647

[123] Moore AD, Anghelescu DL. Emergence Delirium in Pediatric Anesthesia. Paediatr Drugs. 2017;19(1):11–20. 10.1007/s40272-016-0201-5 27798810

[124] van Hoff SL, O’Neill ES, Cohen LC, Collins BA. Does a prophylactic dose of propofol reduce emergence agitation in children receiving anesthesia? A systematic review and meta-analysis. Paediatr Anaesth. 2015;25(7):668–76. 10.1111/pan.12669 25917689

[125] Chandler JR, Myers D, Mehta D, Whyte E, Groberman MK, Montgomery CJ, et al. Emergence delirium in children: a randomized trial to compare total intravenous anesthesia with propofol and remifentanil to inhalational sevoflurane anesthesia. Paediatr Anaesth. 2013;23(4):309–15. 10.1111/pan.12090 23464658

[126] Locatelli BG, Ingelmo PM, Emre S, Meroni V, Minardi C, Frawley G, et al. Emergence delirium in children: a comparison of sevoflurane and desflurane anesthesia using the Paediatric Anesthesia Emergence Delirium scale. Paediatr Anaesth. 2013;23(4):301–8. 10.1111/pan.12038 23043512

[127] Sun L, Guo R. Dexmedetomidine for preventing sevoflurane-related emergence agitation in children: a meta-analysis of randomized controlled trials. Acta Anaesthesiol Scand. 2014;58(6):642–50. 10.1111/aas.12292 24588393

[128] Costi D, Cyna AM, Ahmed S, Stephens K, Strickland P, Ellwood J, et al. Effects of sevoflurane versus other general anaesthesia on emergence agitation in children. Cochrane Database Syst Rev. 2014;9:Cd007084. 10.1002/14651858.CD007084.pub2 PMC10898224 25212274

[129] Tsai PS, Hsu YW, Lin CS, Ko YP, Huang CJ. Ketamine but not propofol provides additional effects on attenuating sevoflurane-induced emergence agitation in midazolam premedicated pediatric patients. Paediatr Anaesth. 2008;18(11):1114–5. 10.1111/j.1460-9592.2008.02593.x 18950343

[130] Hadi SM, Saleh AJ, Tang YZ, Daoud A, Mei X, Ouyang W. The effect of KETODEX on the incidence and severity of emergence agitation in children undergoing adenotonsillectomy using sevoflurane based-anesthesia. Int J Pediatr Otorhinolaryngol. 2015;79(5):671–6. 10.1016/j.ijporl.2015.02.012 25770644

[131] Abu-Shahwan I, Chowdary K. Ketamine is effective in decreasing the incidence of emergence agitation in children undergoing dental repair under sevoflurane general anesthesia. Paediatr Anaesth. 2007;17(9):846–50. 10.1111/j.1460-9592.2007.02298.x 17683402

[132] Lee YS, Kim WY, Choi JH, Son JH, Kim JH, Park YC. The effect of ketamine on the incidence of emergence agitation in children undergoing tonsillectomy and adenoidectomy under sevoflurane general anesthesia. Korean J Anesthesiol. 2010;58(5):440–5. 10.4097/kjae.2010.58.5.440 PMC2881518 20532051

[133] Anghelescu DL, Rakes LC, Shearer JR, Bikhazi GB. Prevention of emergence agitation in seven children receiving low-dose ketamine and propofol total intravenous anesthesia. Aana J. 2011;79(3):238–42. PMC4500836 21751692

[134] Khattab AM, El-Seify ZA. Sevoflurane-emergence agitation: Effect of supplementary low-dose oral ketamine premedication in preschool children undergoing dental surgery. Saudi J Anaesth. 2009;3(2):61–6. 10.4103/1658-354X.57878 PMC2876942 20532105

[135] Heiberger AL, Ngorsuraches S, Olgun G, Luze L, Leimbach C, Madison H, et al. Safety and Utility of Continuous Ketamine Infusion for Sedation in Mechanically Ventilated Pediatric Patients. J Pediatr Pharmacol Ther. 2018;23(6):447–54. 10.5863/1551-6776-23.6.447 PMC6336171 30697129

[136] Roselló Millet P, Muñoz Bonet JI. Techniques and complementary techniques. Intubation, sedation and adaptation to mechanical ventilation. An Pediatr (Barc). 2003;59(5):462–72. 10.1016/s1695-4033(03)78761-x 14588219

[137] Timpe EM, Eichner SF, Phelps SJ. Propofol-related infusion syndrome in critically ill pediatric patients: coincidence, association, or causation? J Pediatr Pharmacol Ther. 2006;11(1):17–42. 10.5863/1551-6776-11.1.17 PMC3468086 23118644

[138] Hayden JC, Breatnach C, Doherty DR, Healy M, Howlett MM, Gallagher PJ, et al. Efficacy of α2-Agonists for Sedation in Pediatric Critical Care: A Systematic Review. Pediatr Crit Care Med. 2016;17(2):e66–75. 10.1097/PCC.0000000000000599 26704469

[139] Miller AC, Jamin CT, Elamin EM. Continuous intravenous infusion of ketamine for maintenance sedation. Minerva Anestesiol. 2011;77(8):812–20. 21730929

[140] Johnson PN, Miller JL, Hagemann TM. Sedation and analgesia in critically ill children. AACN Adv Crit Care. 2012;23(4):415–34. 10.1097/NCI.0b013e31826b4dea 23095967

[141] Umunna BP, Tekwani K, Barounis D, Kettaneh N, Kulstad E. Ketamine for continuous sedation of mechanically ventilated patients. J Emerg Trauma Shock. 2015;8(1):11–5. 10.4103/0974-2700.145414 PMC4335149 25709246

[142] Golding CL, Miller JL, Gessouroun MR, Johnson PN. Ketamine Continuous Infusions in Critically Ill Infants and Children. Ann Pharmacother. 2016;50(3):234–41. 10.1177/1060028015626932 26783355

[143] Tobias JD, Martin LD, Wetzel RC. Ketamine by continuous infusion for sedation in the pediatric intensive care unit. Crit Care Med. 1990;18(8):819–21. 10.1097/00003246-199008000-00004 2379394

[144] Hartvig P, Larsson E, Joachimsson PO. Postoperative analgesia and sedation following pediatric cardiac surgery using a constant infusion of ketamine. J Cardiothorac Vasc Anesth. 1993;7(2):148–53. 10.1016/1053-0770(93)90207-2 8477017

[145] Neunhoeffer F, Hanser A, Esslinger M, Icheva V, Kumpf M, Gerbig I, et al. Ketamine Infusion as a Counter Measure for Opioid Tolerance in Mechanically Ventilated Children: A Pilot Study. Paediatr Drugs. 2017;19(3):259–65. 10.1007/s40272-017-0218-4 28299720

